# World No Tobacco Day — May 31, 2013

**Published:** 2013-05-31

Approximately 6 million deaths related to tobacco use occur each year, including 600,000 in persons who breathe secondhand smoke. Unless trends reverse, by 2030, approximately 8 million persons will die from tobacco use each year. Approximately 80% of these deaths are expected to occur among person living in low-income and middle-income countries (1).

In 1987, the World Health Organization (WHO) created World No Tobacco Day to draw global attention to the health risks of tobacco use. Another important contribution of WHO to conceiving a long-term solution to the global tobacco problem is the development of the WHO Framework Convention on Tobacco Control. The treaty was adopted by the World Health Assembly in 2003 and is one of the most widely embraced treaties in United Nations history (2).

The treaty commits countries to protect the public’s health by adopting various measures to reduce demand for tobacco. One measure of the treaty requires countries to provide widely accessible, comprehensive information regarding the addictive nature, risks, and health threats of exposure to tobacco smoke (3). Antismoking messages in the mass media are one means to accomplish this goal. This issue of *MMWR* includes a review of data from 17 countries, finding an association between awareness of antismoking messages and intention to quit smoking.
